# Effect of Alkali-Free Synthesis and Post-Synthetic Treatment on Acid Sites in Beta Zeolites

**DOI:** 10.3390/molecules25153434

**Published:** 2020-07-28

**Authors:** Kinga Mlekodaj, Joanna E. Olszowka, Venceslava Tokarova, Edyta Tabor, Ales Kasparek, Jana Novakova, Gabriela Stavova, Olga Gonsiorova, Lenka Peliskova, Jiri Brus, Radim Pilar, Petr Klein, Jiri Dedecek

**Affiliations:** 1 J. Heyrovský Institute of Physical Chemistry, Czech Academic Sciences, Dolejškova 3, 18223 Prague, Czech Republic; kingapatrycja.mlekodaj@jh-inst.cas.cz (K.M.); joanna.olszowka@jh-inst.cas.cz (J.E.O.); edyta.tabor@jh-inst.cas.cz (E.T.); radim.pilar@jh-inst.cas.cz (R.P.); petr.klein@jh-inst.cas.cz (P.K.); 2 Jerzy Haber Institute of Catalysis and Surface Chemistry, Polish Academy of Sciences, 30239 Kraków, Poland; 3 Unipetrol Centre for Research and Education a.s., Revoluční 84, 400 01 Ústí nad Labem, Czech Republic; Venceslava.Tokarova@unicre.cz (V.T.); ales.kasparek@unicre.cz (A.K.); jana.novakova@unicre.cz (J.N.); gabriela.stavova@unicre.cz (G.S.); olgs.gonsiorova@unicre.cz (O.G.); lenka.peliskjova@unicre.cz (L.P.); 4 Institute of Macromolecular Chemistry, Czech Academic Sciences, Heyrovského nám. 1888, 16200 Prague, Czech Republic; brus@imc.cas.cz

**Keywords:** beta zeolite, alkali-free synthesis, acid sites, framework Al-Lewis sites, extra-framework Al-Lewis sites, Brønsted acid sites, air calcination, ammonia calcination, de-templating

## Abstract

Beta zeolites with Si/Al around 14 were prepared using three new alkali-free synthesis methods based on the application of amorphous aluminosilicate precursor and calcined in ammonia or air. All samples exhibit structural and textural properties of standard beta zeolite. Comprehensive study by ^27^Al and ^29^Si MAS NMR, together with FTIR adsorption of d_3_-acetonitrile and pyridine were used to characterize the influence of both the synthesis and calcination procedure on the framework Al atoms and related Brønsted and Lewis acid sites. While calcination in ammonia preserves all framework Al atoms, calcination in air results in 15% release of framework Al, but without restrictions of the accessibility of the beta zeolite channel system for bulky pyridine molecules. Terminal (SiO)_3_AlOH groups present in the hydrated zeolites were suggested as a precursor of framework Al-Lewis sites. Surprisingly, the mild dealumination of the air-calcined zeolites result in an increase of the concentration of Brønsted acid sites and a decrease of the total concentration of Lewis sites with the formation of the extra-framework ones.

## 1. Introduction

Zeolite as heterogeneous catalysts are connected not only with the outstanding level of development in refining and petrochemistry in the second half of 20th century [1], but also, they are considered in several new highly required applications as deNOx processes, utilization of biomass and renewables or methane and carbon dioxide [2,3,4]. The position of zeolites as catalysts results from their unique structural properties. Corner-sharing TO_4_ (T = Si, Al) tetrahedra are building mechanically and chemically stable 3D frameworks of nearly 250 structural types of zeolites containing regular channel/cavity systems of molecular size with large surface area ranging hundreds of square meters [5,6]. This well-defined and tunable (by the selection of the zeolite topology) channel systems provides shape selectivity (substrate, product, transition state) for catalytic applications. The isomorphous substitution of Al atoms to the silicate framework introduce a negative charge of the zeolite framework, which has to be balanced by extra-framework cationic species: H^+^ role of Brønsted acidic and transition metal cations role of redox catalytic centers with well-defined but tunable properties and located in the confined space of the zeolite channel system.

Beta zeolite (*BEA topology) [7,8,9] belongs to the group of commercially employed zeolites. Nevertheless, beta zeolite is unique between zeolitic materials as it is the only one large pore (with channels formed by 12 member rings allowing diffusion of a sphere with 5.95 Å diameter) zeolite with 3D system of interconnected channels (more advantageous for catalytic applications than 1D or 2D systems) [10]. While bridging Al-OH-Si Brønsted acid sites predominate in the majority of zeolites in their protonic forms, Al-Lewis acid sites represent a big fraction of acid sites in the H-forms of beta zeolites with a higher concentration of Al atoms in the framework (Si/Al < 20). Al-Lewis acid sites in beta zeolites originate from the spontaneous release of the framework Al atoms during the calcination procedure applied for the removal of organic structure-directing agent (SDA) employed for zeolite synthesis (this type of framework Al-Lewis acid sites can be formed as well by dealumination of the zeolite by steaming) [11] or the perturbation of framework Al T-sites. It was confirmed that even careful template removal that is not accompanied by sample dealumination led to the formation of framework Al-Lewis sites [12]. However, the local arrangement of these framework Al-Lewis sites as well as their precursors in hydrated zeolites and mechanism of their formation was not yet elucidated [13,14]. Nevertheless, the unique combination of acidity and the channels accessibility make beta zeolite highly promising catalyst for reactions catalyzed by Lewis acidity as Friedel–Crafts alkylations, Meerwein–Ponndorf–Verley, and Diels–Alder reactions [15,16].

Standard hydrothermal synthesis to obtain beta zeolites is based on the application of tetraethylammonium cations as a structure-directing agent under the presence of alkali cations (sodium) in the reaction mixture [17,18]. However, to prepare H-form of beta zeolite catalyst for industrial use three post-synthetic steps before final catalyst forming (extruded or pelletized) are required: (i) Removal of organic SDA by the calcination in air which results in NaH-form of the zeolite, (ii) ion exchange of calcined NaH-zeolite with ammonium salt solution to obtain NH_4_-form, and (iii) preparation of H-beta zeolite by the calcination of the NH_4_-form. As the main field of the beta zeolite catalysts application covers reactions catalyzed by acid sites, thus simplification of the post-synthetic treatment leading to H-form of the zeolite is highly demanded in the large-scale production of the catalyst. The most promising way in preparation of beta catalyst lies in the alkali-free synthesis allowing direct extrusion of the as-made zeolitic product with a binder followed by calcination of extrudates to remove organic SDA. The first successful alkali-free synthesis of the beta zeolite with Si/Al around 7 was reported in 1997 [19] and was further developed by G. Wanping group [20,21]. However, industrially applicable and economically feasible alkali-free synthesis of beta zeolite has to meet two requirements: overcome gel formation during synthesis in the presence of electrolytes and minimize the concentration of the organic SDA. The presence of ionic species as electrolytes support gel formation and the behavior of the used reaction mixture plays a key role in further crystallization of the beta zeolite product.

In this paper, three methods of direct alkali-free synthesis of beta zeolites with tetraethylammonium hydroxide as a structure-directing agent are demonstrated. All three methods are based on the solid precursors containing Si- and Al-sources obtained via industrially feasible procedures that minimize the amount of used organic SDA and maximize zeolitic product yield in the batch. Calcined in ammonia and in air alkali-free synthetized beta zeolites were investigated for their acidic properties that are strongly dependent on the de-templating procedure. Calcination in ammonia was already reported as a method to preserve Al atoms in the beta zeolite framework [13]. To elucidate the effect of synthesis procedure and organic SDA removal on the structural and acidic properties of beta zeolites samples calcined in ammonia were compared with samples de-templated in air and characterized with XRD, SEM, low-temperature N_2_ adsorption, ^27^Al and ^29^Si MAS NMR, and FTIR of adsorbed d_3_-acetonitrile and pyridine. It was shown that the application of precursor in the synthesis of alkali-free beta zeolite is highly promising and provides materials with properties corresponding to well-developed *BEA topology. Further, the FTIR study showed that the channel system of all calcined samples is accessible and revealed the presence of Brønsted acid sites, and two groups of framework Al-Lewis sites—Accessible and inaccessible for bulkier pyridine molecules, and finally extra-framework Al-Lewis acid sites. The dealumination of the calcined in air beta zeolites resulted in a decrease of Al-Lewis acid sites and an increase of Brønsted acid sites. Nevertheless, the concentration of accessible for pyridine Al-Lewis acid sites increased with calcination in air and this type of SDA removal provides promising materials for catalytic applications.

## 2. Results

### 2.1. Alkali-Free Synthesis of Beta Zeolites

**Figure 1:**
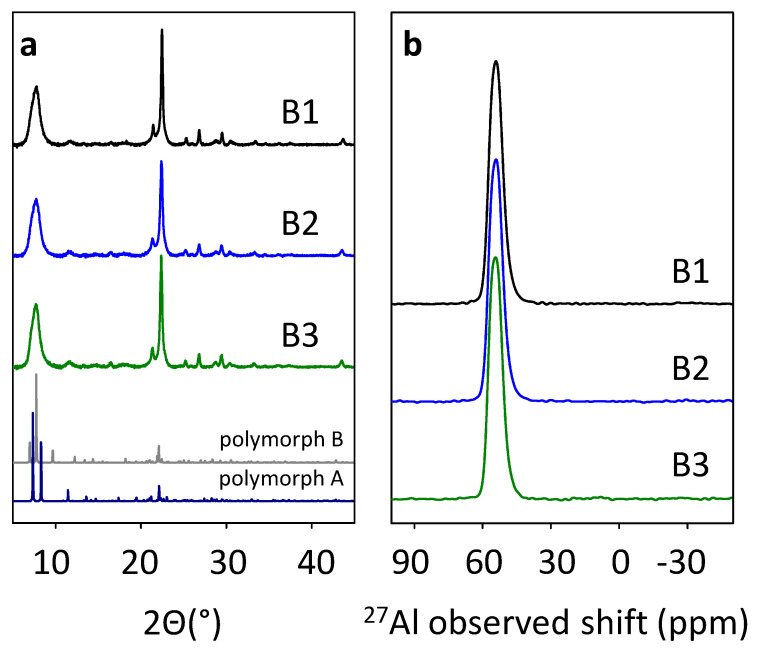
X-ray diffraction patterns of as-synthesized B1–B3 samples together with *BEA polymorphs A and B (**a**) and ^27^Al MAS NMR spectra of as-synthesized B1-B3 samples (**b**).

Three beta zeolite samples were synthesized by an alkali-free hydrothermal approach (in detail described in Section 4. Materials and Methods) from three differently prepared aluminosilicate precursors with the use of TEAOH (tetraethylammonium hydroxide)—As an organic structure-directing agent. Aluminosilicate precursors were employed to avoid technologically undesirable gel formation. The precursors for the synthesis were (i) precipitated from water glass by acidic Al_2_(SO_4_)_3_ solution for sample B1, (ii) spray-dried of 30% silica sol and polyaluminum chloride for the sample B2, and (iii) spray-dried of 3.6% silica sol and Al(OH)_3_ slurry for the sample B3. XRF analysis of as-synthesized B1, B2, and B3 beta zeolites show Si/Al in the range of ca. 14 (see Supplementary Materials, Figure S1). All three samples exhibited well-developed diffraction patterns characteristic for beta zeolite, see Figure 1a in which also patterns of *BEA polymorphs A and B were added for comparison [10]. The ^27^Al MAS NMR spectra of as-synthesized samples showed in Figure 1b exhibit exclusively Al resonance with an observed shift around 55 ppm. This resonance reflects Al atoms in the oxidic environment with tetrahedral coordination and confirms well-developed zeolitic materials of *BEA topology with Al atoms exclusively present in the zeolite framework [22].

### 2.2. Post-Synthetic Treatment—Organic SDA Removal

**Figure 2:**
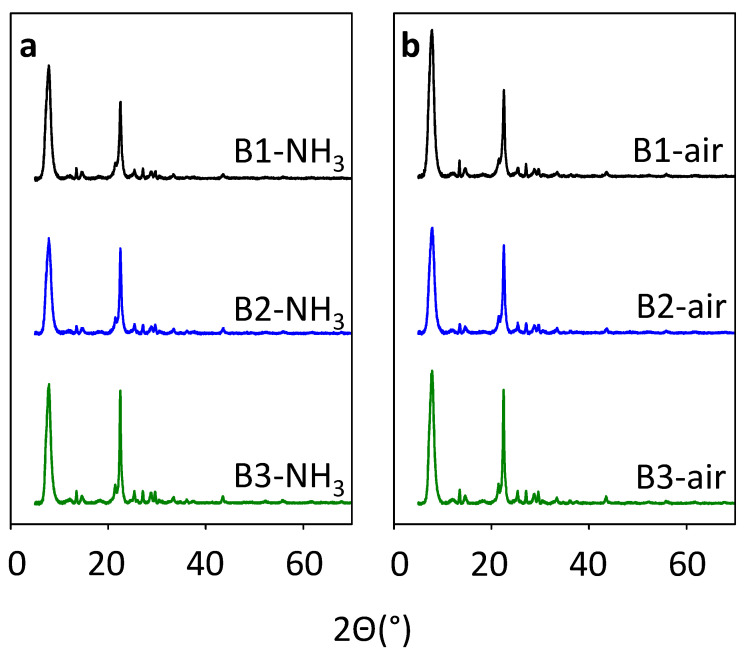
X-ray diffraction patterns of B1–B3 samples calcined in ammonia (**a**) and in air (**b**).

**Figure 3:**
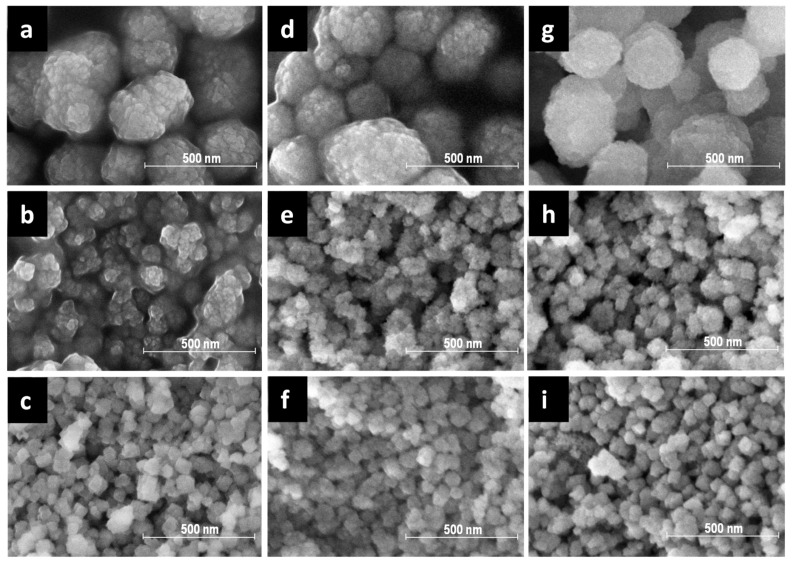
SEM images of B1, B2, and B3 samples as-synthesized (**a**–**c**), calcined in ammonia (**d**–**f**) and in air (**g**–**i**).

Removal of organic SDA by calcination procedure is a tentative step in post-synthetic treatment for the beta zeolite that may lead to the essential release of Al atoms from the framework due to the high amount of organic SDA in the large pore channel system of beta zeolites. The dealumination of the zeolite results in the formation of extra-framework Al species in the channel system. These extra-framework Al species can play the role of Al-Lewis active sites, but also block the transport of reactants in the channel system of zeolite. Furthermore, the excessive dealumination can lead to the collapse of the structure [23,24,25]. In order to study the influence of the organic SDA removal on the structural and textural properties of the prepared via alkali-free synthesis beta zeolites (B1, B2, and B3) the two approaches were used: Standard single-step calcination in air (B1-, B2-, and B3-air), the method generally applied both in the industry and academy, and multi-step calcination in ammonia flow, which aimed at preservation of the zeolite structure from dealumination (B1-, B2-, and B3-NH_3_) [24,25,26,27], that includes consecutive thermal treatment and ion exchange. Despite the calcination procedure, XRD results for B-NH_3_ and B-air series of beta samples confirmed the presence of all diffraction patterns distinctive for beta zeolite see Figure 2 [10]. The observed diffraction peaks at 7 and 22° 2θ proves the presence of both polymorphs B and A respectively of *BEA structure. The visible changes in half-width line of the diffraction patterns of the samples after calcination comparing with as-made zeolites is a consequence of template removal, causing changes in the unit cell parameters. The SEM images of as-synthesized and calcined samples are shown in Figure 3. All images reflect nanocrystallinity of the studied materials without damages after calcination. Crystals of mostly about 10–50 nm size are grown together into rather spherical, roughly ten times larger, agglomerates. The distribution of crystal size is homogenous, without visible defects in crystal habits.

**Figure 4:**
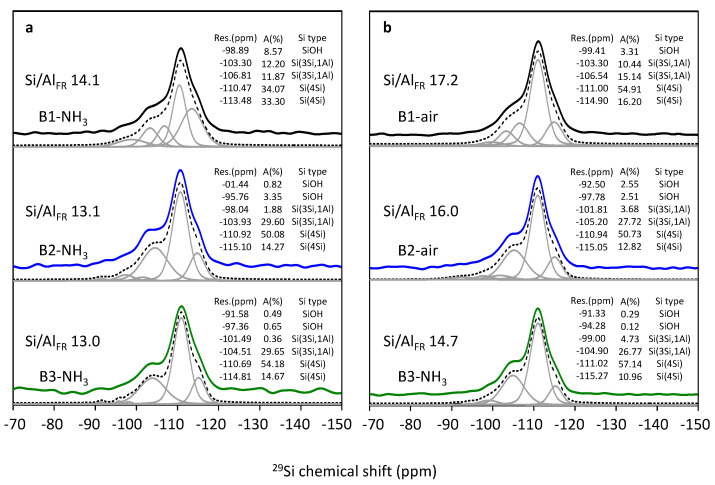
^29^Si MAS NMR spectra of B1-B3 samples calcined in ammonia (**a**) and in air (**b**) solid lines, together with spectra simulation dashed and gray lines.

^29^Si MAS NMR measurements of calcined samples (rehydrated H-form of B-NH_3_ and B-air series) provide quantitative information on the concentration of framework and (indirectly) of extra-framework Al atoms in the zeolite (see Table 1) [28] and were employed to monitor the effect of treatment on the dealumination of the samples. The ^29^Si MAS NMR spectra of B1-B3 samples calcined in ammonia or in air, together with spectra simulations are shown in Figure 4. The spectra correspond to those already reported for beta zeolites [13,14,25,29]. The complex NMR spectra are the result of the combination of a high number of crystallographic Al and Si T-sites in the *BEA structure and the presence of a variety of Si(nSi,4-nAl) atoms in the beta framework. Resonances below −108 ppm reflect Si(4Si) atoms (atoms with exclusively Si neighbors) and resonances between −100–−108 ppm are attributed to Si(3Si,1Al) atoms (Si atoms with one Al neighbor) [23,25,30,31]. All resonances above −100 ppm are attributed to the Si(3Si,1OH) atoms, as follows from the significant increase of their intensity in the ^29^Si CP MAS NMR experiment which amplifies the signal of nuclei in the vicinity of “fixed” protons (see Supplementary Materials, Figure S2). Resonances above −100 ppm characterizing Si(2Si,2Al) atoms in *BEA matrices with Si/Al > 10 were not observed. For detailed attribution of resonances and their intensity, see Figure 4. Al_FR_ in samples B1-NH_3_ and B2-NH_3_ corresponds to the XRF results of as-synthesized ones, while for the sample B3-NH_3_ the observed difference of Al_XRF_ and Al_FR_ represent the release of 7% of framework Al, which is close to the detection limit (ca. 5% for samples with Si/Al 10–15) (Figure 4). On the contrary, dealumination unambiguously occurred during the calcination of all B1-B3 samples in air and reached 13–15% of framework Al atoms, see Table 1.

Analysis of the ^29^Si MAS NMR spectra evidences high concentration of Si(3Si,1OH) atoms in the investigated samples attributed to the terminal Si-OH groups (silanol groups). This is a typical feature of the all studied beta zeolites with the high external surface area due to their small crystal size (ca. 20–50 nm) [32]. It can be concluded, that calcination of B1–B3 samples in ammonia results in the negligible dealumination while the calcination in air results in the release of ca. 15% of Al atoms from the framework. On the other hand, terminal Si-OH groups dominate in B1–B3 samples calcined in ammonia compared to air calcined ones (resonance attribution Figure 4).

**Figure 5:**
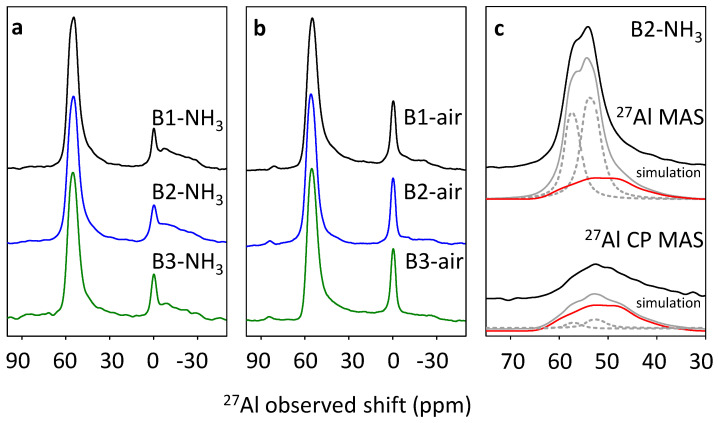
^27^Al MAS NMR spectra of hydrated B1-B3 samples calcined in ammonia (**a**) and in air (**b**). ^27^Al MAS NMR and ^27^Al CP MAS NMR spectra of B2-NH_3_ sample black solid lines, together with spectra simulation (**c**) gray and red lines.

The extra-framework Al atoms occurring in other than tetrahedral coordination (octahedral or penta-coordinated Al) can be detected using the ^27^Al MAS NMR experiment. However, beta zeolites represent an exception in this respect and signal characteristic for octahedral Al atoms observed for calcined and rehydrated H-form of beta zeolites is present even if exclusively framework Al atoms are present in the sample [26,27]. The ^27^Al MAS NMR of the B1–B3 beta zeolite samples calcined in ammonia or in air are presented in Figure 5a,b. The spectra of all calcined samples exhibit signal centered around 55 ppm of Al atoms in the tetrahedral environment, however, they are more complex in comparison with those of as-synthesized samples (Figure 1b). In addition, the resonances below 5 ppm characteristic for octahedrally coordinated Al atoms are also present in ^27^Al MAS NMR spectra [25,31]. The tetrahedral signal exhibits maxima with observed shifts around 54 and 58 ppm and a significant shoulder at the lower shift region.

Simulation of the spectrum (see Figure 5c) shows the presence of two narrow Gaussian-like resonances with isotropic shifts 55.8 and 59.1 ppm (and Cq values 2.5 and 2.3 MHz respectively) and a broad asymmetric one with isotropic shift 62.5 ppm (and Cq value 5.5 MHz). The presence and shape of the broadened resonance were confirmed by the ^27^Al low-energy cross-polarization experiment (^27^Al MAS NMR) performed on the Na-form of the beta zeolites to enhance the visibility of terminal framework (SiO)_3_AlOH atoms. The narrow bands with isotropic shifts 55.8 and 59.1 ppm correspond to bands with isotropic shifts around 55 and 58 ppm well known for beta zeolites [26,33], and reflect Al atoms in a highly symmetric tetrahedral environment of the framework T sites in the beta zeolite matrix. Differences in the values of the isotropic shifts in the studied beta zeolites can be explained by the change of the geometry of the T site or by the presence of a second Al atom in the vicinity of Al atom [34,35]. The isotropic shift 62.5 ppm of the broadened resonance evidenced that this resonance indicated Al atoms in a tetrahedral oxidic environment. Nevertheless, significant quadrupolar broadening reflected in Cq = 5.5 MHz suggests a less symmetric environment of the corresponding tetrahedral Al atom. Similar resonances (isotropic shift 58.9–62.4 ppm and Cq 4.8–5.3 MHz) were reported for chabasite and ferrierite topologies and attributed to Al atoms in the terminal framework (SiO)_3_AlOH groups at the external surface of the zeolite crystals [35]. The amount of the corresponding Al atoms varies from 20 to 30% of the total aluminum (calculated from ^27^Al-NMR data, details in the Supplementary Materials) among all B1–B3 samples calcined in both ammonia and air. The attribution of the broadened resonance at 62.5 ppm to framework Al in the spectra of all studied H-beta zeolites was confirmed by the ^27^Al CP MAS NMR experiment, see Figure 5c. This experiment determines well the Al atoms in close proximity of protons, thus the intensity of the signal of Al atoms in the terminal framework (SiO)_3_AlOH groups is enhanced in comparison to the narrow resonances at 55.8 and 59.1 ppm in ^27^Al CP MAS NMR (Table S1) [36]. The sharp resonance at 0 ppm in the ^27^Al MAS NMR spectrum (Figure 5b) of B1–B3 samples calcined in air is attributed to the extra-framework octahedral Al atoms (relative intensity of this signal spans from 11 to 13%) confirmed by ^29^Si NMR experiment, which shows the release of 10–15% of framework Al atoms. The ^27^Al MAS NMR spectra of B1–B3 samples calcined in ammonia exhibit a broad band between 10 and –30 ppm and a narrow band at 0 ppm. While the broad band may reflect unambiguously the presence of framework octahedral Al atoms, the narrow resonance can reflect either framework Al atoms or a negligible amount of extra-framework Al atoms.

**Figure 6:**
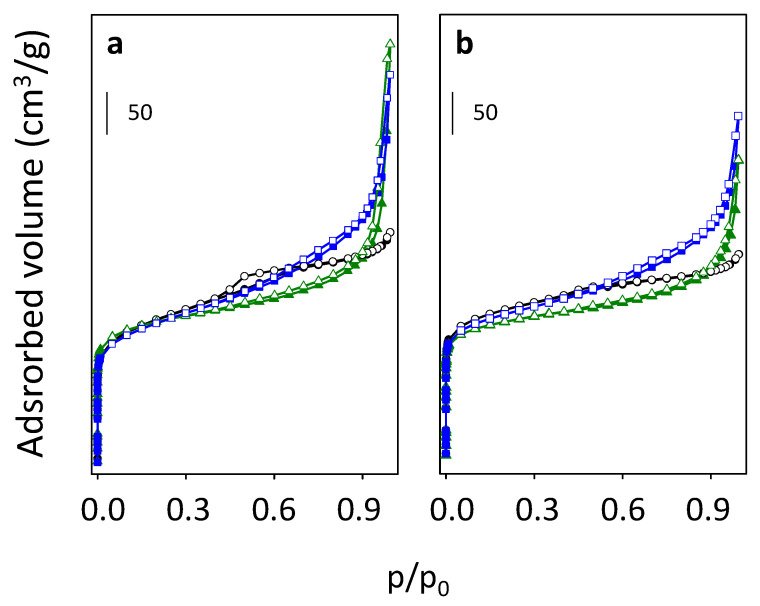
Low-temperature N_2_ adsorption (full symbols) and desorption (empty symbols) isotherm registered for B1 (black), B2 (blue) and B3 (green) samples calcined in ammonia (**a**) and in air (**b**).

Accessibility of the microporous channel system of the all beta zeolites after removal of the SDA was characterized using low-temperature N_2_ adsorption (Figure 6). 

N_2_ adsorption results for samples B2 and B3, after both calcination procedures (ammonia and air) revealed well-developed hysteresis in contrast to both B1 samples (B1-NH_3_ and B1-air), where the hysteresis is not shaped. It confirmed that the presence of the hysteresis is directly connected with the methods of precursor preparation, as B2 and B3 samples were prepared from spray-dried precursors leading to aggregates formation, in contrast to B1 which was obtained from a wet precipitated precursor. Higher values of the surface areas for air calcined B1, B2, and B3 zeolites than for the equivalents calcined in ammonia can suggest partial extraction of framework Al atoms. However, samples calcined in ammonia exhibited higher values of external surface areas unlike samples de-templated in air (Figure 6, Table 2, and Table S2). This higher external surface area values may be connected with repeated thermal stress and mechanical mixing during consecutive ion exchange process, and thus leading to the opening of cracks in-between agglomerate building crystals. All beta zeolites possess a significant fraction of mesopores with diameters around 12.5 (see Supplementary Materials, Figure S3). 

### 2.3. FTIR Studies

#### 2.3.1. Accessibility of the Channel System

**Figure 7:**
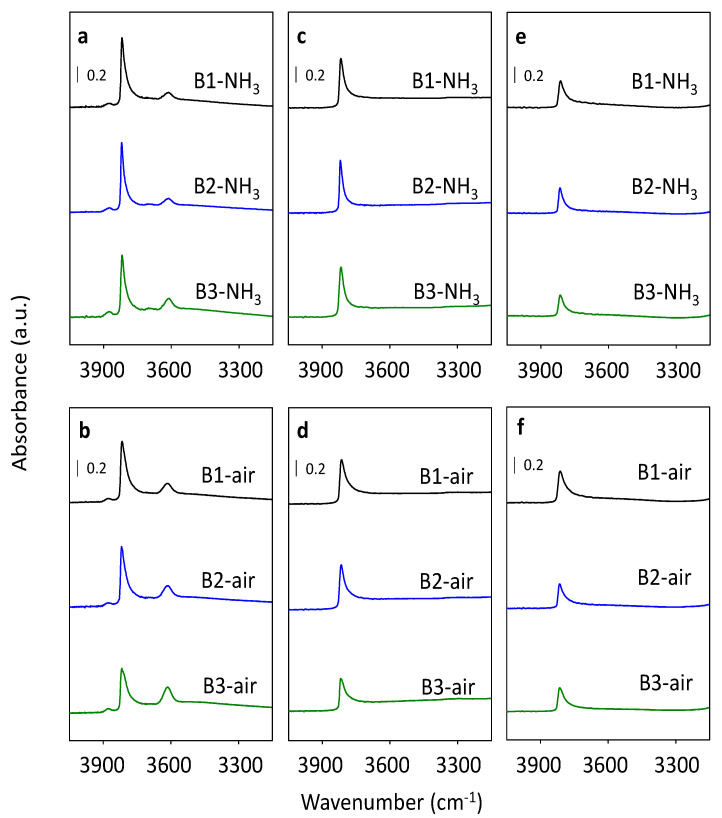
The effect of d_3_-acetonitrile (**c**,**d**) and pyridine (**e**,**f**) adsorption in the region of OH vibrations of B1–B3 evacuated samples calcined in ammonia (**a**) and in air (**b**).

In order to characterize the accessibility, the nature and the concentration of acid sites in B1, B2, and B3 samples that undergo organic SDA removal by calcination in the ammonia or in air, the FTIR spectroscopy studies using two molecules d_3_-acetonirtile and pyridine were applied [37,38,39]. Both molecules were selected as a probe for spectroscopic characterization of the acid sites in the zeolite, but they differ in geometry: Linear and easy diffused d_3_-acetonirtile, and cyclic molecule-pyridine. Prior to the adsorption, all samples were evacuated at 450 °C for 3 h under vacuum. In order to characterize the accessibility of beta channels system, the FTIR spectra were analyzed in the OH vibration region (4000–3200 cm^−1^) before and after adsorption of d_3_-acetonitrile or pyridine (Figure 7) [23,25,37,40]. The strong Brønsted acid sites, which are known to interact with weak base probes (d_3_-acetonitrile or pyridine) are distributed inside the beta zeolite channel system. Thus, the interaction of all these sites with probe molecules can be used as evidence of accessibility of the channel system. The detailed procedure for calculation of the concentration of the particular acid sites in studied samples detected by both d_3_-acetonitrile or pyridine is described below. FTIR spectra of all evacuated samples exhibited the four types of vibrations of OH groups: Al-OH groups at 3780 cm^−1^, terminal Si-OH groups at 3745 cm^−1^, and Brønsted acid sites Si-OH-Al at 3605 cm^−1^ accompanied by a broad perturbed bridging Si-OH-Al groups ranging 3600–3200 cm^−1^ (Figure 7a,b). The band attributed to Brønsted bridging Si-OH-Al groups is less represented at the FTIR spectra of samples calcined in ammonia in comparison to air calcined samples. However, the band at 3745 cm^−1^ attributed to Si-OH groups is more intense for all B1–B3 ammonia calcined samples. Under the adsorption of both weak base probe molecules (d_3_-acetonitrile and pyridine), the band at 3605 cm^−1^ representing Brønsted acid sites disappeared (Figure 7c–f). This result confirmed that the whole channel system of the B1, B2, and B3 zeolites calcined in both ammonia and in air is accessible to d_3_-acetonitrile and pyridine molecules. 

#### 2.3.2. Brønsted and Lewis Acidity

**Figure 8:**
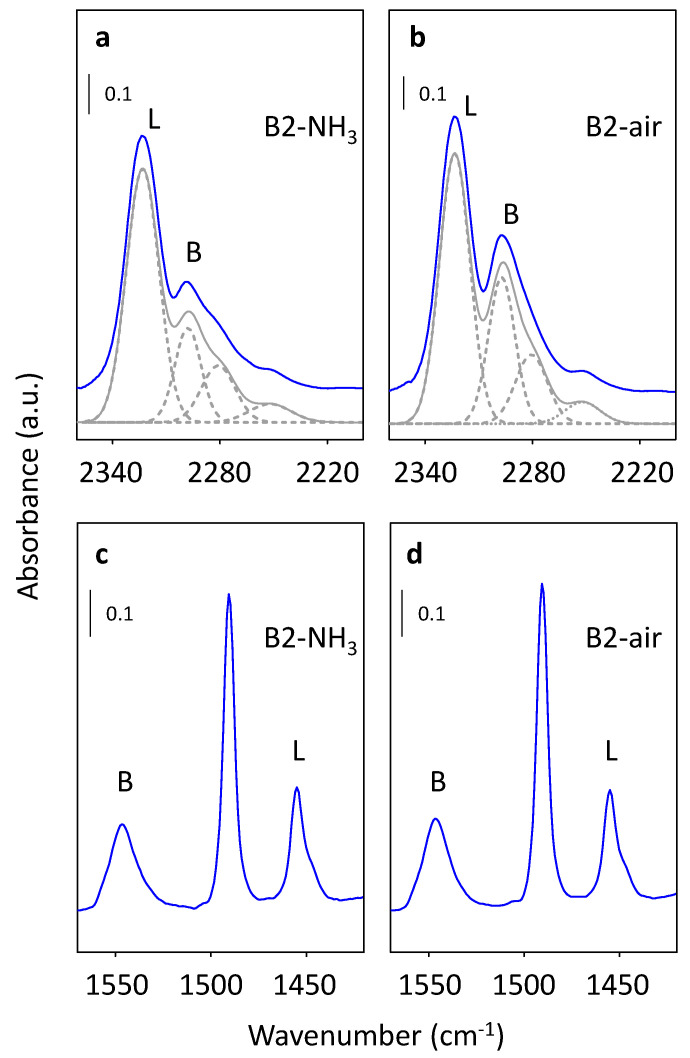
FTIR spectra of adsorbed d_3_-acetonitrile for dehydrated H-forms of calcined in ammonia (**a**) and air (**b**) B2 sample together with deconvolution of the spectra (gray lines) and after adsorption of pyridine (**c**,**d**).

d_3_-Acetonitrile as a probe molecule was used for the detection of all accessible acid sites present in zeolites. The adsorption of d_3_-acetonitrile is manifested in the vanishing of the band at 3605 cm^−1^ assigned to Brønsted acid site (Figure 7) with simultaneous formation of a complex band between 2220 and 2360 cm^−1^ in the FTIR spectra of B1, B2, and B3 samples calcined in ammonia or in air, see Figures S4 and S5 in Supplementary Materials. For deconvolution of the band between 2220 and 2360 cm−1 previously established methodology was used, recognizing the four subspectra corresponding to d_3_-acetonitrile adsorbed on (i) Si-OH-Al Brønsted acid sites at 2297 cm^−1^, (ii) Al-Lewis acid sites at 2325 cm^−1^, (iii) terminal Si-OH groups on 2275–2282 cm^−1^, and (iv) the surface on 2250–2252 cm^−1^ [37,40]. The deconvoluted FTIR spectra of adsorbed d_3_-acetonitrile and pyridine were shown as an example for sample B2 calcined in ammonia or in air (Figure 8). The concentration of Brønsted and Lewis acid sites was calculated as described in Section 4. Materials and Methods and are gathered in Table 3 using already published extinction coefficients. The FTIR studies of d_3_-acetonitrile adsorption confirmed the influence of the calcination methods on the concentration of Brønsted acid sites, which are roughly 30% less populated in B1–B3 ammonia calcined samples in comparison with air calcined analogs. However, the calcination does not significantly influence the concentration of the Lewis acid sites accessible for the d_3_-acetonitrile molecule.

The FTIR spectra of adsorbed pyridine on B1, B2, and B3 samples de-templated in ammonia or in air revealed the presence of the band at 3605 cm^−1^ assigned to Brønsted bridging Si-OH-Al groups, and the subsequent appearance of the bands at 1545 cm^−1^ and 1445–1460 cm^−1^ attributed to pyridine interaction with the Lewis and Brønsted acid sites, respectively. The concentration of Brønsted and Lewis acid sites calculated from adsorption of pyridine (described in Section 4. Materials and Methods) using already published extinction coefficients is summarized in Table 3 [37,40]. Similarly, to FTIR results of d_3_-acetonitrile adsorption, the concentration of Brønsted acid sites is lower for samples treated with ammonia than calcined in air. However, the total concentration of Brønsted acid sites detected by pyridine is slightly higher for both series of beta samples than the concentration of Brønsted acid sites estimated from d_3_-acetonitrile adsorption. The observed discrepancy in Brønsted acid sites concentration is up to 15% of a method error. However, due to the necessity of the d_3_-acetonitrile spectra simulation in comparison to the straightforward analysis of pyridine adsorption spectra, in further discussion Brønsted acid sites concentration values from pyridine adsorption will be used. Analogously the calcination procedure of the B1–B3 beta samples represents the same trend observed already for d_3_-acetonitrile adsorption on all studied beta samples, there is no essential influence on the concentration of the Lewis acid sites detected from the pyridine adsorption. The results gathered in Table 3 showed that the Lewis acid sites concertation in B1-B3 analogs calcined in ammonia or in air revealed changes up to 26%. On the other hand, the significantly lower fraction of the Lewis acid sites is accessible for the pyridine molecule in contrast with linear d_3_-acetonitrile.

## 3. Discussion

### 3.1. Tailored Alkali-Free Synthesis of Beta Zeolite

Avoiding gel formation in the alkali-free systems for hydrothermal synthesis of beta zeolite is not trivial and as it was shown, the properties of the used precursor play a key role in the successful crystallization of the zeolitic product. Precipitated wet precursors made from water glass and acidic Al_2_(SO_4_)_3_ solution known from the successful synthesis of alkali-containing ZSM-5 and beta [41] are easy to handle as they behave rather as a precipitate than as a gel (synthesis of sample B1). The precursor prepared from 30% silica sol by spray-drying together with an Al source leads to gel formation during hydrothermal synthesis. To avoid it this system required large volumes of starting mixtures to obtain solid-dry powder precursor (synthesis of sample B2). For the third precursor, 30% silica sol was used as a starting material for spray-drying together with ionic Al source, but this time oxalic acid and fluorides were added as complex-forming agents for Al cations to block their ionic form and prevent silica oligomers from gelation (synthesis sample B3). Structural studies (XRD, ^27^Al MAS NMR) of as-synthesized samples (B1-B3) revealed that all three alkali-free synthetic approaches result in well-developed (high crystallinity, phase purity) beta zeolites with structural parameters typical for *BEA topology and corresponding to beta zeolites synthesized by standard hydrothermal synthesis with the use of alkali (Figure 1). Thus, the application of an aluminosilicate precursor can be suggested as an efficient method for the prevention of the gel formation during the alkali-free synthesis of beta zeolite. Moreover, in all synthesis described in this paper TEAOH/Si ratio of ca. 0.3 is significantly lower in comparison to previously reported alkali-free syntheses of beta zeolites which required TEAOH/Si in the range 0.5–0.7 [19,21].

### 3.2. Effect of Post-Synthetic Treatment on the Beta Zeolite Structure

As the structure of beta zeolite is known to be sensitive to calcination, which can influence the character of Al-related acid centers by the transfer of the part of Al in framework T-sites to the extra-framework Al species, all three beta zeolites were calcined in ammonia or in air. ^29^Si MAS NMR experiments show a negligible or very low release of Al atoms from the framework for studied B1–B3 zeolites calcined in ammonia (Figure 4). This, together with results of X-ray diffraction (Figure 2) and SEM (Figure 3) confirms that B1-B3-NH_3_ samples represent materials with preserved crystallinity and significant structure degradation was not observed. NMR studies showed that calcination in air led to a noticeable dealumination of samples (10–15% of framework Al atoms, see Table 1). Nevertheless, the channel system of both ammonia and air calcined B1-B3 samples is accessible to N_2_ molecules (Figure 6). Moreover, FTIR studies of adsorbed probe molecules on evacuated samples of both B-NH_3_ and B-air series confirmed the accessibility of zeolitic channels also to bigger molecules as d_3_-acetonitrile and pyridine (Figure 7). Thus, the mild dealumination (up to 15% framework Al atoms) in B-air samples did not result in the decrease of crystallinity, and restraint of the channel system accessibility.

FTIR analysis of the OH vibration region (4000–3200 cm^−1^) for all B1-B3 samples after calcination revealed a higher concentration of terminal Si-OH groups for samples after calcination in ammonia, which may suggest higher perturbation of the framework (Figure 7). A significantly higher concentration of Si atoms bearing -OH groups (ca. 2.5 times) in samples B1-B3 calcined in ammonia in comparison to their analogs calcined in air was also confirmed by ^29^Si NMR experiment (Figure 4) despite a negligible amount of extra-framework Al atoms of the former. Thus, some perturbation of the zeolitic framework not connected with the leaching of Al atoms from the framework had to occur. This perturbation could be connected with the presence of the Si-OH groups which may lead to the formation of framework Al-Lewis sites observed in ammonia calcined beta samples. 

### 3.3. Framework Al Atoms in De-Templated and Rehydrated Beta Zeolite

Comparison of the FTIR studies on the adsorption of d_3_-acetonitrile and pyridine (Figure 8) on B-NH_3_ and B-air series of samples revealed that d_3_-acetonitrile allows the investigation of whole present Al-Lewis acid sites, while > 50% of Al-Lewis acid sites are not accessible for pyridine (Table 3). Thus, we may distinguish two groups of Al-Lewis sites in the B1-3-NH_3_ samples with the exclusive presence of framework Al atoms: Accessible and inaccessible for pyridine adsorption. Moreover, according to ^27^Al MAS NMR three types of framework Al atoms can be distinguished in all calcined and rehydrated beta samples: (i) Predominating framework Al atoms in the highly symmetric tetrahedral (SiO)_4_ environment, (ii) framework octahedral Al atoms, representing 15–25% of all Al atoms which were already suggested to be connected with framework Al-Lewis sites, and (iii) terminal (SiO)_3_AlOH atoms on the zeolite surface representing ca. 25% of all Al atoms, up to now reported only as a minor fraction for ferrierite and chabasite structures [35,42]. In the paper of Brus et al. the terminal (SiO)_3_AlOH atoms in zeolites were suggested based on the FTIR and ^27^Al NMR studies of dehydrated beta samples to be a precursor of surface (SiO)_3_Al atoms with Lewis acidity. Note that these (SiO)_3_Al atoms can be formed by the dehydration of the terminal (SiO)_3_AlOH atoms without perturbation of the framework, for details see [35,42]. The concentration of accessible for pyridine molecules framework Al-Lewis sites in B1-B3 ammonia calcined samples fits well with the concentration of (SiO)_3_AlOH atoms in hydrated samples from ^27^Al MAS NMR (Table S1). Thus, it can be suggested that terminal (SiO)_3_Al atoms represent accessible for pyridine framework Al-Lewis sites in studied beta samples without a significant presence of extra-framework Al atoms (B-NH_3_ series) and (SiO)_3_AlOH atoms may be suggested as their precursors. Note, that the high concentration of these accessible for pyridine framework Al-Lewis sites can be connected with the large external surface area of nanosized beta samples, however, the already reported ^27^Al MAS NMR spectra of beta zeolites [23,24,25,43] suggest that significant presence of the (SiO)_3_AlOH atoms, not usual for beta zeolites, is connected with the synthesis procedure. In the case of samples with extra-framework Al species, accessible for pyridine Al-Lewis sites correspond to both framework and extra-framework Al-Lewis sites. While the inaccessible framework Al-Lewis sites can be tentatively attributed to the already reported [12,44] framework Al-Lewis sites connected with the perturbation of the zeolite framework.

### 3.4. Effect of Post-Synthetic Treatment on the Beta Zeolite Acidity

**Figure 9:**
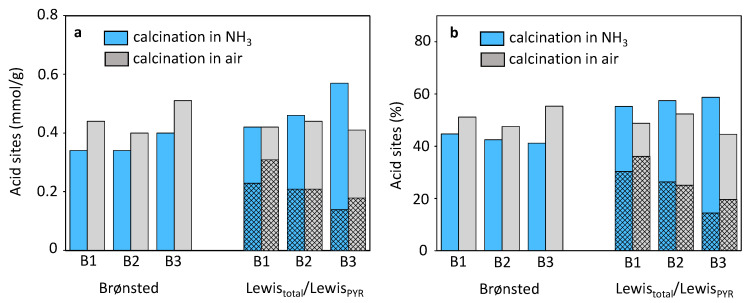
Comparison of the Brønsted and Lewis acid sites concentration (Lewis_total_ derived from FTIR of acetonitrile adsorption; Brønsted and chequered Lewis_PYR_ derived from pyridine adsorption) in mmol/g (**a**) and as percentage value (**b**) in B1–B2 beta zeolites.

The quantitative evaluation of the FTIR experiments of pyridine adsorption clearly shows that the concentration of the Brønsted bridging Si-OH-Al groups increased with dealumination, see Figure 9 and Table 3. As it was mentioned in the introduction part, Brønsted acid sites are formed by bridging framework Al-OH-Si sites, while a new type of Al-Lewis site attributed to extra-framework Al atoms were formed during calcination in air inducing the dealumination. It can be suggested that the observed increase of Brønsted acid sites concentration is created at the expense of the framework Al-Lewis acid site in the beta zeolite [12,23,24,25]. As it is observed for B1-NH_3_ and B2-NH_3_ samples, where 2.25 Al atoms correspond to one inaccessible for pyridine framework Al-Lewis site (See Supplementary Materials, Table S3). Release of the one Al atom from the cooperating Al atoms forming framework Al-Lewis acid site (inaccessible for pyridine) contributes to the formation of extra-framework Al-Lewis acid site, while the residual framework Al-atom takes part in the formation of Brønsted acid site. The detailed structure of extra-framework Al-Lewis acid sites in zeolites was not yet clearly described and thus, the number of corresponding Al atoms is not known. Moreover, the number of Al atoms required for the formation of accessible and inaccessible for pyridine framework Al-Lewis sites varies. It may be expected that the relative increase of Brønsted acid sites after dealumination has to be followed by a relative decrease of Al-Lewis site concentration (Figure 9b). However, the dealumination of B1 and B3 samples in air results in the decrease in the total amount of Al-Lewis sites. This can be explained by the fact that several Al atoms cooperate on the formation of one extra-framework Al-Lewis site.

On contrary to the decrease of the total amount of Al-Lewis acid sites, the concentration of Al-Lewis acid sites accessible for pyridine and thus representing possible active sites for the transformation of bulkier molecules did not change with the dealumination for B2-air sample, and for B1-air and B3-air samples increased. Consequently, samples de-templated by technologically less demanding calcination in air can be suggested to be more promising in the application for catalysts used in Lewis acidity catalyzed reactions than samples with minimized perturbation of Al atoms.

According to the data in Table 3 the increase of the concentration of accessible for pyridine Al-Lewis acid sites between B1 and B3 beta samples despite the calcination procedure is up to 70% (from 0.12–0.15 mmol to 0.23–0.31 mmol). On contrary the increase of the accessible for pyridine Al-Lewis acid sites connected with the de-templating procedure reaches up to 35% (Figure 9). Therefore, the effect of the synthesis procedure on the concentration of accessible for pyridine Al-Lewis sites is more pronounced for various synthesis procedure than the calcination manner. Without a doubt, the effect of synthesis on the number of accessible for pyridine Al-Lewis acid sites occurs due to the formation of (SiO)_3_AlOH atoms representing precursors for terminal framework Al-Lewis acid sites. Thus, alkali-free synthesis of beta zeolites using precursors brings, besides technological advantages, also a high amount of accessible for bulkier molecules (such as pyridine) Al-Lewis acid sites.

## 4. Materials and Methods

### 4.1. Synthesis of the Zeolites

Three beta zeolites B1, B2, and B3 with Si/Al ca. 14 were prepared by alkali-free synthesis procedures using organic structure-directing agents (SDA).

#### 4.1.1. Synthesis of Beta Zeolite B1

The precursor was precipitated from water glass solution (175 g of SiO_2_/dm^3^ and 52 g of Na_2_O/dm^3^) in a stirred continuous reactor by acidic Al_2_(SO_4_)_3_ solution (277 g of SO_4_^2−^/dm^3^ and 35.5 g of Al_2_O_3_/dm^3^) at pH 8 to have molar Si/Al ratio 14.5. The slurry was filtered, washed with 1 M ammonium nitrate solution and distilled water to remove sodium cations, then dried at 100 °C. The as-prepared precursor (3100 g, 28.4% of dry matter) was mixed with 1393 g of 43.7% TEAOH solution and 10.8 g of seeding crystals of the commercial beta zeolite were added. The mixture was stirred 30 min then transferred into an anchor stirred autoclave for hydrothermal synthesis at 135 °C for 72 h. The molar TEAOH/Si ratio was 0.3. After the hydrothermal synthesis, the slurry was acidified by diluted nitric acid to pH 9, next filtered, washed with distilled water, and dried at 105 °C overnight.

#### 4.1.2. Synthesis of Beta Zeolite B2

The precursor was prepared by spray-drying of silica sol and polyaluminum chloride with complex-forming agents. A mixture of NH_3_ stabilized 30% silica sol (3600 g), oxalic acid dihydrate (180 g), polyaluminum chloride solution PAX-18 (KEMIRA, 8.65% Al, 446.4 g), and ammonium hydrogen fluoride (41.04 g) with 1 dm^3^ of distilled water was spray dried. The product was dried at 250 °C overnight. Next, 750 g of the as-prepared precursor was mixed with 35% TEAOH solution (1575.54 g), distilled water (1672 g), and 7.5 g of the seeding commercial beta zeolite crystals. Hydrothermal synthesis ran in an anchor stirred autoclave at 140 °C for 96 h. The molar TEAOH/Si ratio was 0.3. After the synthesis, the slurry was acidified by nitric acid diluted up to pH 9, then filtered and washed with distilled water, finally dried at 105 °C overnight.

#### 4.1.3. Synthesis of Beta Zeolite B3

The precursor was prepared by spray-drying of diluted silica sol and aluminum hydroxide slurry. A mixture of 3.6% silica sol (6 dm^3^) and Martigloss (35.92 g, a slurry containing 65.15% of aluminum hydroxide in water provided by Martinswerk GmbH) was spray dried. Then, 6000 g of the as-prepared precursor was mixed with 35% TEAOH solution (10,001 g), distilled water (9280 g), and 60 g of the commercial seeding beta crystals. TEAOH/Si ratio was 0.3. Hydrothermal synthesis ran in an anchor stirred autoclave at 140 °C for 96 h. After the synthesis, the slurry was acidified by diluted to pH 9 nitric acid, next filtered and washed with distilled water, finally dried at 105 °C overnight.

### 4.2. Organic SDA Removal

Organic SDA was removed from the synthesized beta zeolites by two different procedures. By calcination in ammonia or in air.

#### 4.2.1. Calcination in Ammonia (Samples B1-, B2-, and B3-NH_3_)

Dried as-synthesized samples were exchanged three times with 0.5 M sodium nitrate solution for 24 h. All samples were then filtered, washed with demineralized water, and dried. The zeolites were calcined in ammonia stream with a ramp 2 °C/min to 100 °C and 90 min dwell, further the temperature was increased to 420 °C with the same ramp and 120 min dwell. After calcination, samples were once more ion-exchanged with 0.5 M sodium nitrate solution three times for 24 h. Next calcination occurred in air flow with a ramp 2 °C/min to 100 °C and 90 min dwell, further the temperature was increased to 520 °C with the same ramp and 120 min dwell. Finally, zeolites were exchanged with 1 M ammonium nitrate solution three times for 2 h, filtered, washed, and dried. The last calcination step was done in an oven in air flow with a ramp 0.6 °C/min to 540 °C and 480 min dwell.

#### 4.2.2. Calcination in the Air (Samples B1-, B2-, and B3-air)

Dried as-synthesized zeolites were calcined in an oven in air flow with a ramp 0.6 °C/min to 540 °C and 480 min dwell.

All calcined samples (B-NH_3_ and B-air series) were ion-exchanged to Na- or NH_4_-forms (3 × 24 h, RT, 0.1 dm^3^ of the solution/1 g of the zeolite, 0.5 M NaNO_3_ and 1M NH_4_NO_3_, respectively). Na-forms of studied samples were used for ^27^Al CP MAS NMR, structural characterization, and adsorption experiments, while NH_4_-forms were used for FTIR studies. ^27^Al MAS NMR experiments were also performed for as-synthesized samples, and ^27^Al and ^29^Si MAS NMR were measured for B1, B2, B3 samples after calcination in both ammonia and air in their H-forms.

### 4.3. Characterization Methods

Chemical analysis of as-synthesized zeolites was done by X-ray fluorescence using spectrometer BRUKER AXS S8 Tiger with a 5% error.

The crystalline structure of as-synthesized and calcined ion-exchanged samples was determined by X-ray diffraction on BRUKER AXS D8 Advance.

Scanning electron microscopy (SEM) of calcined ion-exchanged samples was performed on a JEOL JSM-5500LV apparatus.

Adsorption properties, surface area and porosity of zeolites were characterized by measurements of nitrogen sorption and desorption isotherms at 77 K on Quantachrome Autosorb iQ. Before the analysis the samples were heated to 395 °C for 19 h at pressure 0.13–0.15 Pa. For BET surface area calculation, the range p/p_0_ from 0.007 to 0.05 was used for all three calcined in air beta zeolites and sample B3-NH_3_, whereas for ammonia calcined B1 and B2 the upper value of p/p_0_ range 0.01 was used. Total pore volume was calculated from p/p_0_ range 0.994–0.995. Micropore and mesopore volume together with total pore volume were calculated by the NLDTF method with a model for silica with cylindrical pores. For external surface determination and micropore volume evaluation, t-analysis was used. Thus, micropore volume was determined by two methods (t-analysis and NLDTF analysis), total pore volume and surface area were also calculated by two methods (BET and NLDTF).

The combination of ^27^Al and ^29^Si MAS NMR was employed to analyze the state of aluminum in the investigated samples. ^27^Al and ^29^Si MAS NMR spectra of the fully hydrated samples (as-synthesized and H-form), packed in ZrO_2_ rotors, were measured on a Bruker Avance III HD 500 WB/US (11.7 T) spectrometer using 4 mm double-resonance probe at a rotation frequency of 10–12 kHz and 7 kHz, respectively. The ^27^Al MAS NMR spectrum was acquired after 128 scans delayed by 2 s using π/12 pulse width of 0.9 μs with high-power proton decoupling. Low-power ^27^Al cross-polarization (CP) spectrum allowing excitation of components with large quadrupolar splitting was collected using a standard cross-polarization pulse sequence with 500 μs contact time, a relaxation delay of 2 s. The number of 75.776 scans was employed for collecting the ^27^Al CP MAS NMR spectrum of Na-forms of zeolites with an acceptable signal-to-noise ratio. The rotation frequency of the sample was 7.5 kHz. The ^27^Al isotropic chemical shifts were referenced to the aqueous solution of Al(NO_3_)_3_.

^29^Si MAS NMR single pulse spectrum was collected after 1840 cycles using a π/2 excitation pulse width of 4 μs and a recycle delay of 20 s. The cross-polarization (CP) pulse sequences with a 50% ramp CP pulse and 1500 μs contact time, a relaxation delay of 7 s and a number of scans of 4096 was employed for collecting the ^29^Si CP MAS NMR spectrum. The ^29^Si isotropic chemical shifts were referenced to trimethylsilyl ester of the double-four ring octameric silicate (Q_8_M_8_). The analytical simulation of the ^27^Al MAS NMR and ^29^Si MAS NMR spectra were performed using the DM fit software [45].

The percentage of Al atoms in the terminal framework (SiO)_3_AlOH groups was estimated by using ^27^Al MAS NMR spectra simulations.

In the absence of resonances of Si(2Si,2Al) atoms in the ^29^Si MAS NMR spectra, framework aluminum content (Si/Al_FR_) can be estimated using Equation (1):

Si/Al_FR_ = I/0.25 I_1_ (1)

where I stands for the total intensity of the ^29^Si NMR signal in the single-pulse experiment and I_1_ denotes the intensity of the NMR line corresponding to the Si(3Si,1Al) atoms [46].

FTIR spectra were recorded using Nicolet 6700 spectrometer equipped with liquid nitrogen cooled detector (resolution 2 cm^−1^, 128 scans/min). All spectra were recorded at room temperature.

In order to characterize the accessibility, the nature, and concentration of Lewis and Brønsted acid sites the FTIR spectroscopy studies of two probe molecules d_3_-acetonirtile, and pyridine were applied. The B-NH_3_ and B-air series of samples were prepared and analyzed as self-supported pallets placed in the cuvette allowing evacuation, heating, and dosing of gases. Prior to the adsorptions of both d_3_-acetonirtile and pyridine, and spectral measurements all studied samples were evacuated at 450 °C for 3 h under dynamic vacuum. d_3_-Acetonitrile and pyridine were degassed by freeze-pump-thaw cycles.

Adsorption of 1.3 kPa of d_3_-acetonitrile on evacuated samples took place at RT for 30 min and was followed by evacuation at RT for 30 min. The bands of adsorbed d_3_-acetonitrile were deconvoluted into subspectra and approximated by Gaussian profiles using Origin 8.1 software based on their previously established positions [40] assigned to d_3_-acetonitrile interactions with: Al-Lewis acid sites in the region 2323–2329 cm^−1^, Brønsted acid sites in the region 2293–2299 cm^−1^, residual Na cations or terminal Si-OH groups in the region 2275–2282 cm^−1^, and in the region 2250–2252 cm^−1^ for surfaced adsorbed d_3_-acetonitrile. To calculate the concentration of Lewis and Brønsted acid sites following extinction coefficient values were used: η_L_ and η_B_ 3.62 and 2.05 cm µmol^−1^ respectively [40].

1.3 kPa of pyridine was adsorbed on evacuated samples at 150 °C for 30 min, followed by evacuation at 150 °C for 10 min. To calculate the concentration of the acid sites, the integral intensities of the bands at 1545 cm^−1^ and 1445–1460 cm^−1^ attributed to pyridine interaction with the Lewis and Brønsted acid sites respectively together with the values of corresponding extinction coefficients η_L_ and η_B_ 2.22 and 1.67 cm µmol^−1^ [37,40]. The additional band at 1490 cm^−1^ in the FTIR spectra of adsorbed pyridine on B1-B3 samples calcined in ammonia or air cannot be attributed to the single acid site [47].

## 5. Conclusions

The alkali-free synthesis of beta zeolite allows preparation of directly applicable in catalytic processes acidic H-forms of beta zeolites by avoiding two post-synthetic steps: ion exchange of de-templated samples to its NH_4_-form and subsequent transformation of NH_4_-beta to the protonic one by thermal treatment. However, the alkali-free synthesis of the beta zeolite was previously described [19], the application of aluminosilicate precursor requires significantly lower (up to 50%) SDA/Si ratio, and may lead to Si-richer materials (Si/Al 13–15 instead of Si/Al 7–8). The synthetic approach based on the utilization of amorphous aluminosilicate precursors not only prevents the formation of technologically disadvantageous gel during the synthesis but also maximize synthesis yields. Described in this paper three unique alkali-free synthetic approaches provide well crystalline nano-sized beta materials with the channel system accessible after organic SDA removal by both calcination in air and careful de-templating based on treatment in the ammonia stream and consecutive ion exchange.

FTIR studies of the adsorption of two probe molecules with different geometry d_3_-acetonitrile and pyridine on studied beta samples allow to distinguish besides Brønsted acid sites, three groups of Al-related Lewis acid sites (i) Extra-framework Al-Lewis sites, well known for beta zeolites and formed by Al atoms released from the framework, which are at least partially available for pyridine: (ii) Framework Al-Lewis sites accessible both for d_3_-acetonirtile and pyridine, which may be formed from the terminal (Si-O)_3_-AlOH groups present in hydrated zeolites, known up to now to represent a minor fraction of acid sites in ferrierite and chabasite zeolites. This type of Lewis acid sites is confirmed for beta zeolites for the first time and represents a noticeable fraction of acid sites in the samples. (iii) Framework Al-Lewis acid sites accessible for d_3_-acetonirtile but not for pyridine. Although the restricted accessibility of Al-Lewis acid sites for pyridine adsorption was not yet reported, this type of acid sites was known for beta zeolites with Al atoms exclusively preserved in the framework after the de-templating procedure. The amount of accessible for pyridine framework Al-Lewis sites may vary from both the applied synthetic approach and post-synthetic treatment aimed at SDA removal. Surprisingly, the dealumination of the beta zeolites during calcination in air results in the increase of the concentration of Brønsted acid sites and the decrease of the total concentration of Al-Lewis sites (estimated from FTIR studies of adsorbed d_3_-acetonitrile). Nevertheless, the increase in the concentration of Al-Lewis sites accessible for pyridine can be connected with dealumination.

Concluding, alkali-free synthesis of beta zeolites using amorphous aluminosilicate precursors represents a promising, technology-friendly way for the preparation of H-form of beta zeolite with structural and textural properties corresponding to materials prepared using standard synthetic approaches. De-templating of all studied beta samples in air results in mild dealumination of zeolites however, for dealuminated samples, the concentration of Al-Lewis acid sites accessible for bulkier probe molecule as pyridine does not decrease in comparison to samples calcined in ammonia. Thus, presented in this paper beta zeolites synthesized by alkali-free approach may undergo technology-friendly single step calcination in air resulting in samples relevant for the hydrocarbon processing Lewis acid sites and may become easily applicable catalysts.

## Figures and Tables

**Table 1:** Results of chemical analysis (XRF) and concentration of framework and extra-framework Al, Si/Al_FR_ and Al_FR_ estimated from ^29^Si MAS NMR, Al_EF_ estimated as a difference between Al_XRF_ and Al_FR_.

Sample	Si/Al_FR_	Al_XRF_ (mmol/g)	Al_FR_ (mmol/g)	Al_EF_ (mmol/g)
B1-NH_3_	14.1	1.00	1.00	0.00
B2-NH_3_	13.1	1.13	1.13	0.00
B3-NH_3_	13.0	1.21	1.13	0.08
B1-air	17.2	1.00	0.87	0.13
B2-air	16.0	1.13	0.96	0.17
B3-air	14.7	1.21	1.05	0.16

**Table 2:** Results of low-temperature nitrogen adsorption and desorption isotherms.

	B1-NH_3_	B1-Air	B2-NH_3_	B2-Air	B3-NH_3_	B3-Air
BET surface area, m^2^/g	671	739	659	718	690	697
total pore volume, cm^3^/g	0.45	0.41	0.74	0.66	0.80	0.58
micropore volume *, cm^3^/g	0.17	0.23	0.17	0.21	0.23	0.24
external surface area *, m^2^/g	265	176	254	206	136	110

* Values from t-analysis.

**Table 3:** Results of d_3_-acetonitrile (10 Torr, 30 min adsorption/desorption at RT) and pyridine (10 Torr, 30 min adsorption and 10 min desorption at 150 °C) adsorption (FTIR spectra normalized using wafer thickness).

	d_3_-Acetonitrile (mmol/g)		Pyridine (mmol/g)	
Sample	Brønsted	Lewis	Brønsted	Lewis
B1-NH_3_	0.24	0.42	0.34	0.23
B2-NH_3_	0.25	0.46	0.34	0.21
B3-NH_3_	0.32	0.57	0.40	0.14
B1-air	0.35	0.42	0.44	0.31
B2-air	0.37	0.44	0.40	0.21
B3-air	0.46	0.41	0.51	0.18

## Supplementary Materials

The following are available online, Figure S1: XRF spectra of as-synthesized B1–B3 samples, Figure S2: ^29^Si MAS (black lines) and ^29^Si CP MAS NMR (red lines) spectra of hydrated B1–B3 samples calcined in ammonia (a) and in air (b), Figure S3: Pore size distribution in samples B1–B3 calcined in ammonia and in air calculated from N_2_ desorption, Figure S4: FTIR spectra of dehydrated H-forms of B1–B3 samples calcined in ammonia (a–c) and in air (d–f) after adsorption of d_3_-acetonitrile together with deconvolution of the spectra (grey lines), Figure S5: FTIR spectra of dehydrated H-forms of B1-B3 samples calcined in ammonia (a) and in air (b) after adsorption of pyridine, Table S1: The percentage of Al atoms in terminal framework (SiO)_3_AlOH groups, Table S2: Results of low-temperature nitrogen adsorption and desorption isotherms. Values from NLDTF analysis, Table S3: Concentration of acid sites in B1-NH_3_ and B2-NH_3_.
