# Supplementary material for: Effect of Alkali-Free Synthesis and Post-Synthetic Treatment on Acid Sites in Beta Zeolites
Source: Molecules. 2020 Jul 28;25(15):3434. doi: 10.3390/molecules25153434 (PMC7435978; doi:10.3390/molecules25153434)
Supplement: Supplementary file 1 [file molecules-25-03434-s001.pdf]

molecules

Supplementary Materials

XRF

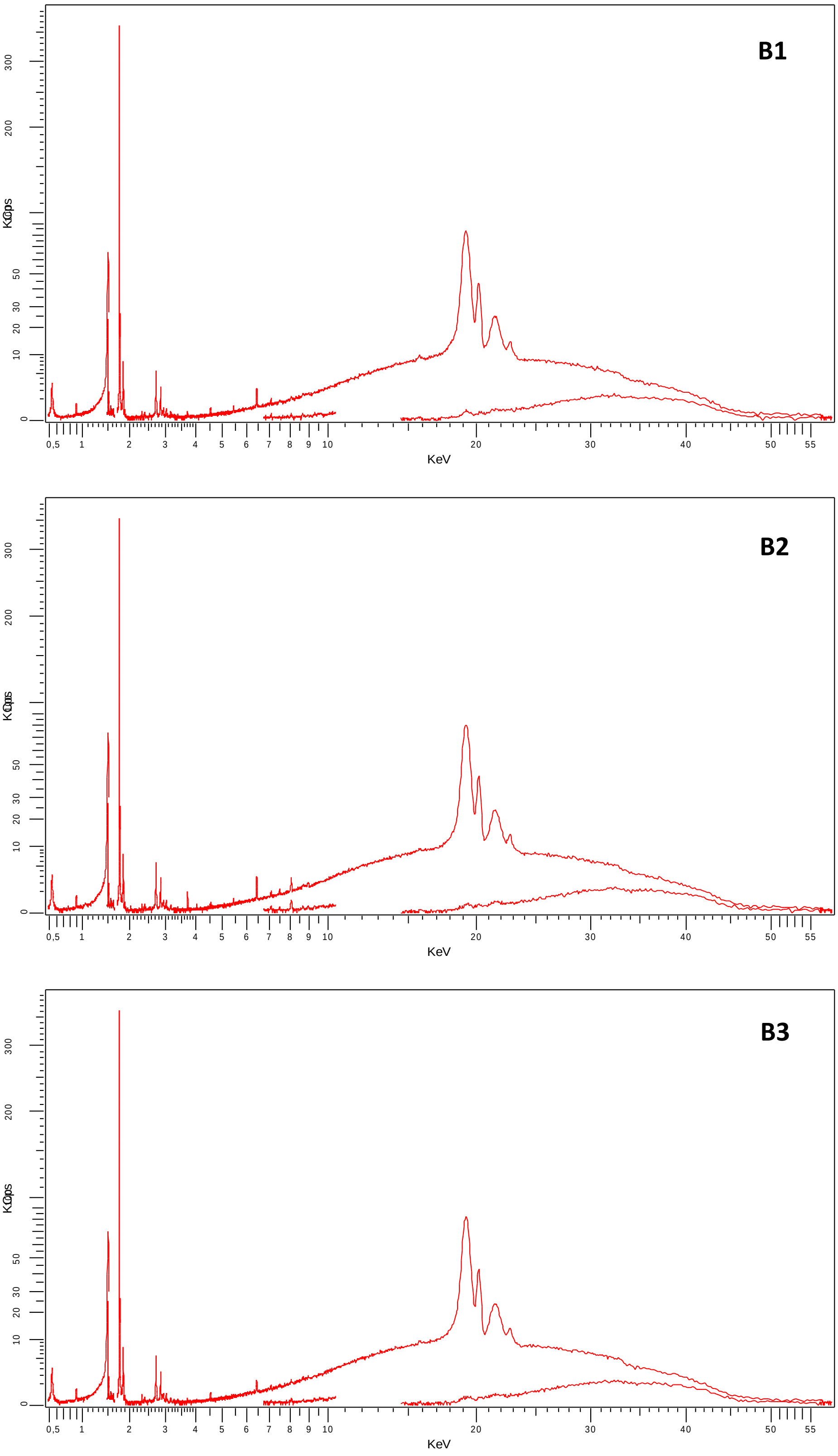

Figure S1. XRF spectra of as-synthesized B1-B3 samples.

^{29}Si MAS NMR

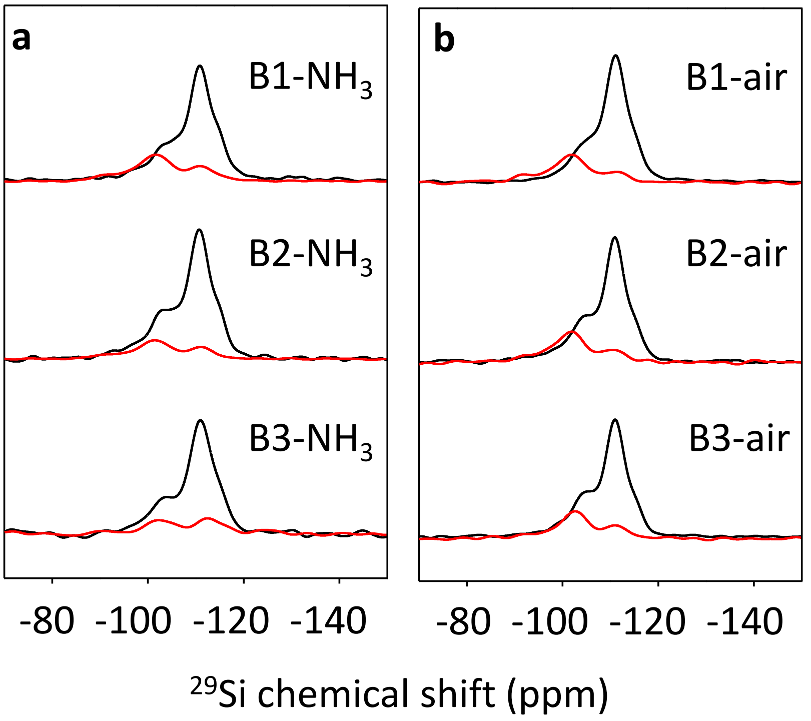

Figure S2. ^{29}Si MAS (black lines) and ^{29}Si CP MAS NMR (red lines) spectra of hydrated B1-B3 samples calcined in ammonia (**a**) and in air (**b**).

^{27}Al MAS NMR

Table S1. The percentage of Al atoms in terminal framework $(SiO)_3AlOH$ groups.

Sample	$Al_{(SiO)3AlOH}$
B1-NH_3	25
B2-NH_3	25
B3-NH_3	25
B1-air	30
B2-air	20
B3-air	25

Low-temperature N_2 adsorption and desorption

Table S2. Results of low-temperature nitrogen adsorption and desorption isotherms. Values from NLDTF analysis.

	B1-NH_3	B1-air	B2-NH_3	B2-air	B3-NH_3	B3-air
total surface area#, m^2/g	937	1071	929	1068	1004	1053
total pore volume#, cm^3/g	0.42	0.38	0.62	0.57	0.64	0.49
micropore volume#, cm^3/g	0.17	0.22	0.17	0.21	0.21	0.23
mesopore volume#, cm^3/g	0.24	0.15	0.40	0.32	0.34	0.22

Pore size distribution

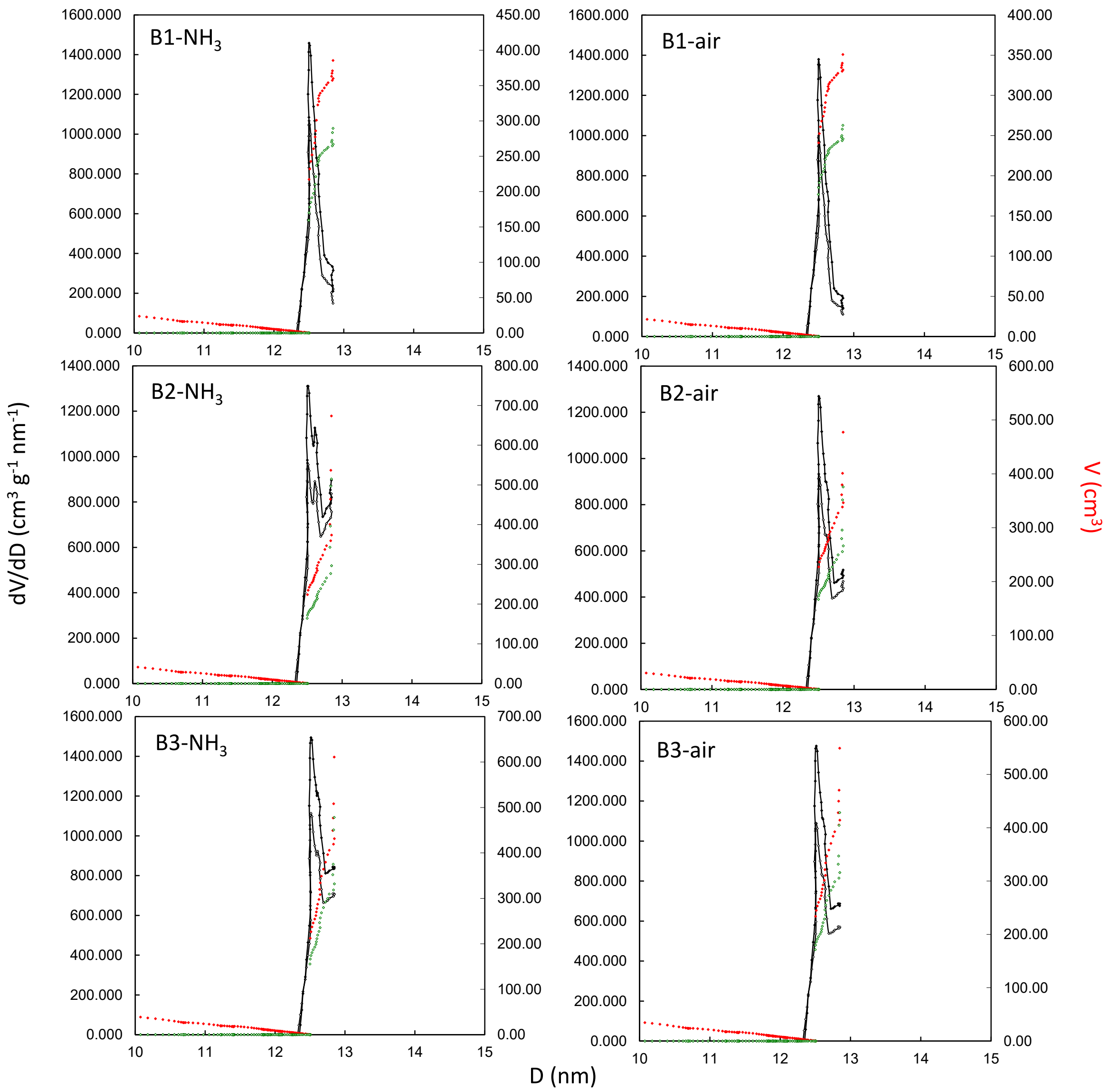

Figure S3. Pore size distribution in samples B1-B3 calcined in ammonia and in air calculated from N_2 desorption.

FTIR experiments

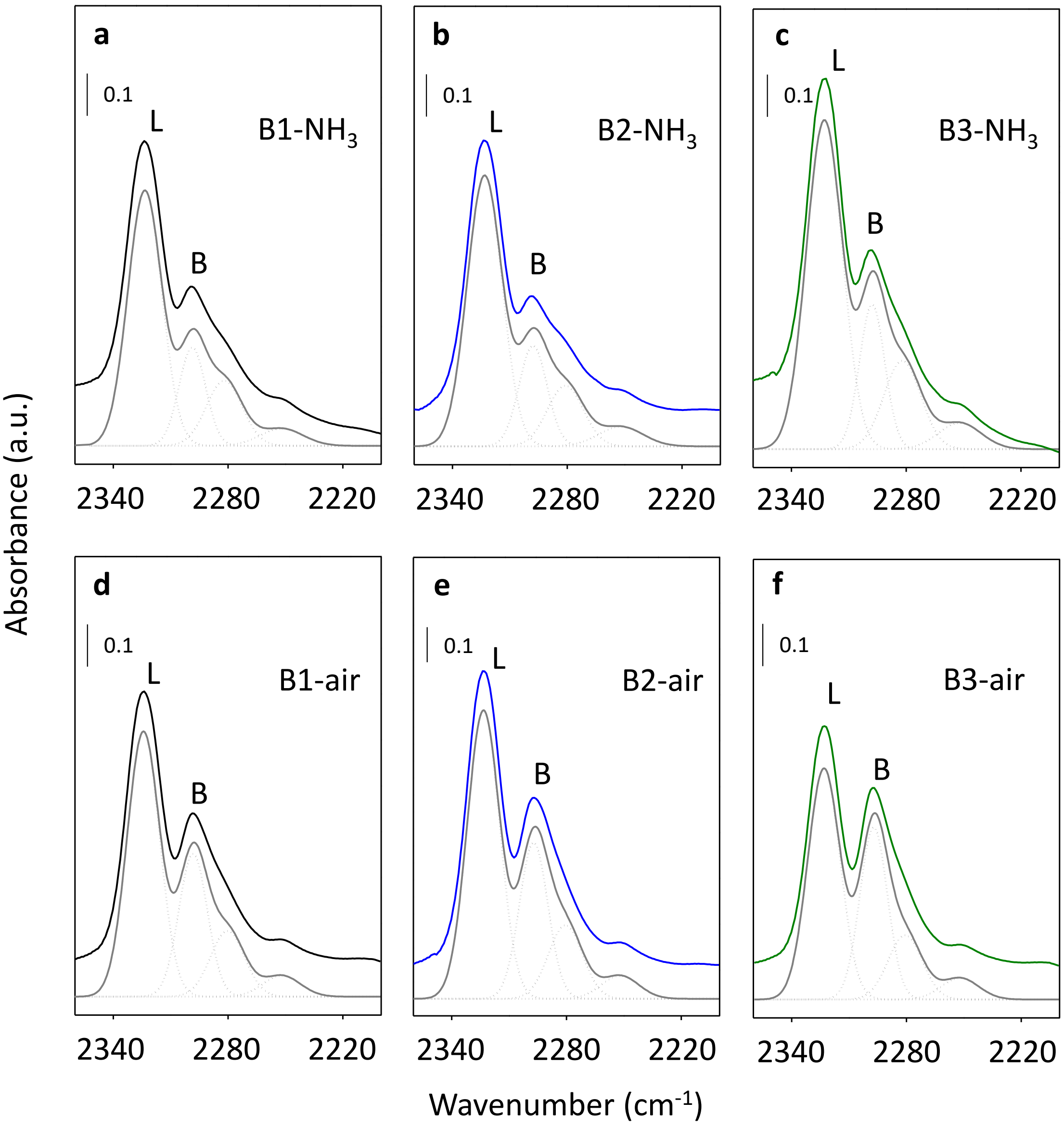

Figure S4. FTIR spectra of dehydrated H-forms of B1-B3 samples calcined in ammonia (**a-c**) and in air (**d-f**) after adsorption of d_3-acetonitrile together with deconvolution of the spectra (grey lines).

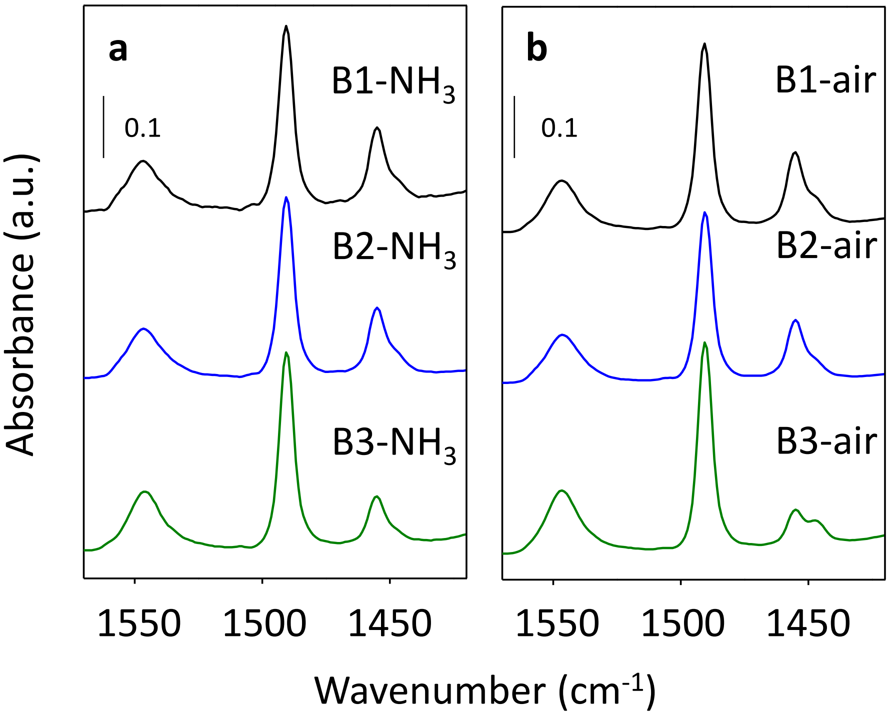

Figure S5. FTIR spectra of dehydrated H-forms of B1-B3 samples calcined in ammonia (**a**) and in air (**b**) after adsorption of pyridine.

Calculation of the number of Al atoms corresponding to Al-Lewis site not accessible to pyridine

Table S3. Concentration of acid sites in B1-NH_3 and B2-NH_3

**Sample**	**d_3-Acetinitrille Lewis [mmol/g]**	**Pyridine Brønsted [mmol/g]**	**Pyridine Brønsted [mmol/g]**	**XRF Al [mmol/g]**
B1-NH_3	0.42	0.34	0.23	1.0
B2-NH_3	0.46	0.34	0.21	1.13

One Brønsted Al-OH-Si bridging site corresponds to one Al atom.

In this paper we suggest that one terminal $(Si\text{-}O)_3\text{-}AlOH$ precursor of framework Al-Lewis site accessible to pyridine corresponds to one framework Al-Lewis site accessible to pyridine.

Number of framework Al-Lewis site inaccessible to pyridine represents the difference between the total number of Al-Lewis sites in the sample with exclusively framework Al atoms and number of framework Al-Lewis site accessible to pyridine:

$$[Lewis_{NOT}] = [Lewis_{ACCE}] - [Lewis_{PYR}]$$

Number of Al atoms corresponding to framework Al-Lewis site inaccessible to pyridine (X) is then

$$X = ([Al_{XRF}] – 1x[Brønsted] – 1x[Lewis_{PYR}]) / [Lewis_{NOT}]) = ([Al_{XRF}] – 1x[Brønsted] – 1x[Lewis_{PYR}]) / ([Lewis_{ACCE}] - [Lewis_{PYR}])$$

B1: $X = (1.00 – 0.34 – 0.23) / (0.42 - 0.23) = 0.43/0.19 =$ **2.26**

B2: $X = (1.13 – 0.34 – 0.21) / (0.46 – 0.21) = 0.58 / 0.25 =$ **2.32**

[Al$_{XRF}$] – concentration of Al atoms in the sample, [Brønsted] – concentration of Brønsted Al-OH-Si bridging sites, [Lewis$_{PYR}$] – concentration of Lewis sites reviled by pyridine adsorption, [Lewis$_{ACCE}$] - concentration of Lewis sites reviled by d$_3$-acetonitrile adsorption, [Lewis$_{NOT}$] – concentration of Lewis sites not accessible for pyridine.
